# The relationship between on-water and off-water performance tests in elite canoe slalom athletes

**DOI:** 10.3389/fspor.2025.1589085

**Published:** 2025-05-19

**Authors:** Matej Vajda, Felix Krupa, Jaylene Pratt, Monika Škáchová, Milan Kováč, Ján Cvečka, Ján Busta

**Affiliations:** ^1^ Sport and Performance Consulting, Bratislava, Slovakia; ^2^ Faculty of Physical Education and Sports, Comenius University, Bratislava, Slovakia; ^3^ Auckland University of Technology, Auckland, New Zealand; ^4^ The Faculty of Sports Science and Health, MBU, Banská Bystrica, Slovakia; ^5^ Národné Športové Centrum, Bratislava, Slovakia; ^6^ Incubator of Kinanthropology Research, Faculty of Sports Studies, Masaryk University, Brno, Czechia; ^7^ No Borders, Civic Association (CA), Holíč, Slovakia; ^8^ Faculty of Physical Education and Sport, Charles University, Prague, Czechia

**Keywords:** canoe slalom, performance testing, physical fitness, whitewater, off-water testing

## Abstract

**Purpose:**

This study aimed to evaluate the relationship between on- and off-water performance tests in canoe slalom.

**Methods:**

A total of 34 elite canoe slalom athletes, who competed in one of the following categories, namely K1 men (K1M, *n* = 9), K1 women (K1W, *n* = 8), C1 men (C1M, *n* = 9), or C1 women (C1W, *n* = 8), volunteered for the study. On-water testing consisted of two flat water tests: sprints with turns to both sides (SBS; 2 × 15 m shuttle sprints) and an all-out shuttle test (12 × 15 AOT; 12 × 15 m shuttle sprints). Off-water testing included anthropometric analyses, power output in bench press, pull measurement, and 3 × 200 m performance on a kayak ergometer. Each athlete completed testing over two consecutive days.

**Results:**

The results showed a significant relationship between the on-water tests (SBS/AOT) and body weight (kg, *r* = 0.472/0.478), body fat (%, *r* = 0.451/0.445), Pmax bench press (W, *r* = 0.748/0.705), Pmax bench pull (W, *r* = 0.704/0.693), relative Pmax bench press (W/kg, *r* = 0.735/0.663), relative Pmax in bench pull (W/kg, *r* = 0.727/0.700), ergo best 200 m (s, *r* = 0.851/0.884), ergo best mean 200 m (W, r = 0.902/0.922), and ergo 3 × 200 m total time (s, *r* = 0.842/0.884), determined using the Pearson correlation coefficient.

**Conclusion:**

Based on the identified relationships, we recommend regular monitoring of the physical fitness levels of canoe slalom athletes using the described off-water tests. These tests can help identify the strengths and weaknesses of athletes, enabling coaches to optimize the training process.

## Introduction

Canoe slalom is one of the canoeing sports that has been a regular part of the Olympic Games since Barcelona 1992. The competition is organized on natural or artificial whitewater courses specifically built for canoe slalom (1). Typical race duration varies in the range between 90 and 120 s and is dependent on the course characteristics, such as water level difficulty, the length of the course, and the number of gates and their position (2). According to the rules of the International Canoe Federation (ICF), the course must consist of a minimum of 18 gates and a maximum of 25 gates, of which six or eight must be upstream gates (at least three for each side). During the competition, the athlete’s goal is to navigate a boat through a combination of upstream and downstream gates. The slalom competition includes four events, namely, men's kayak (K1M), men's canoe (C1M), women's kayak (K1W), and women's canoe (C1W), and athletes are allowed to start in both kayak and canoe events (1).

Currently, for water testing, many coaches and performance analysts in canoe slalom regularly use flat water tests that contain turning maneuvers. In competition runs, these maneuvers are needed for the negotiation of the upstream gates and the combination of downstream gates. These require higher force than straightforward paddling, and energy expenditure is higher during flatwater paddling with turning maneuvers compared with straight-line paddling (3). These tests were first described and analyzed by Baláš et al. (4), who combined turning maneuvers with paddling over 40, 80, and 200 m. The authors revealed that high-performance athletes at the international level had better results in every distance compared to the national level athletes, and the tests had moderate to excellent reliability [inter class correlation (ICC) = 0.680–0.929]. Based on the findings of Baláš et al. (4), Vajda and Piatriková (5) designed and described flatwater tests with turning maneuvers that exhibited a strong relationship with performance in canoe slalom on different water terrain grades (*r* = 0.638–0.909). In addition, these tests demonstrated high reliability, with an ICC of 0.96–0.98.

Physical fitness is an important part of canoe slalom performance (6, 7). According to the systematic review by Messias et al. (8), there are limited numbers of studies focused on analyzing physical fitness in canoe slalom athletes. Only two studies, Bielik et al. (9, 10), have analyzed physical fitness parameters such as strength and power in bench press/pull, VO_2max_, or power output on paddling ergometers. However, these studies were designed to compare parameter levels between canoe slalom medalists from the World European junior or U23 championship and to compare canoe slalom and canoe sprint athletes, respectively. There is no evidence in the literature about the relationship between physical fitness parameters and on-water performance in canoe slalom.

This study was designed using limited evidence, the authors' experiences as canoe slalom coaches, and discussions with other coaches and experts working in canoe slalom worldwide. Our goal was to provide a better understanding of the current role of physical fitness in elite canoe slalom athletes.

Therefore, this study aimed to examine on-water and off-water performance parameters and to identify their interrelationship in canoe slalom athletes.

## Methods

### Subjects

A total of 34 canoe slalom athletes who competed in one of the following categories, namely K1M (*n* = 9), K1W (*n* = 8), C1M (*n* = 9), or C1W (*n* = 8), volunteered for the study. The general characteristics of the athletes are presented in Table 1. The athletes included in the study were members of the National U18 or the U23 team and met the performance criteria for elite/international-level athletes according to the specifications of McKay et al. (11).

**Table 1:** General and anthropometric characteristics of the athletes.

Parameters		K1 women	C1 women	K1 men	C1 men
		(*n* = 8)	(*n* = 8)	(*n* = 9)	(*n* = 9)
Age (years)	X ± SD	19.57 ± 2.29	19.13 ± 1.82	18.66 ± 1.73	19.00 ± 2.17
	95% CI	17.64–21.49	17.60–20.65	17.33–19.99	17.32–20.67
Body height (m)	X ± SD	1.66 ± 0.07	1.65 ± 0.07	1.80 ± 0.07	1.82 ± 0.03
	95% CI	1.60–1.72	1.58–1.71	1.75–1.86	1.79–1.85
Body weight (kg)	X ± SD	57.05 ± 5.60	57.38 ± 7.76	74.81 ± 6.49	74.37 ± 6.50
	95% CI	52.36–61.73	50.90–63.87	69.81–79.80	69.38–79.37
Body fat (%)	X ± SD	16.00 ± 5.22	16.23 ± 2.95	9.13 ± 3.14	9.17 ± 1.37
	95% CI	11.62–20.37	13.76–18.71	6.71–11.51	8.12–10.23

The Ethical Committee of the Faculty of Physical Education and Sports at Comenius University in Bratislava approved the study in accordance with the ethical standards of the Helsinki Declaration. The athletes were fully informed about the nature and potential risks of all procedures before providing written informed consent. The study was conducted with the support of grant VEGA no. 1/0573/22.

### Procedures

All the athletes underwent testing over two consecutive days. On-water testing was performed on the first day. On the second day, off-water testing was carried out in cooperation with the Slovak National Sport Center. All the athletes had previous experience with all the tests and were familiar with the testing procedures. The athletes were asked to obtain enough sleep on the preceding night, not to participate in intense exercise in the 24 h before testing, and not to eat in the 2 h before the measurement session. Data were collected over three seasons during regular post-season testing (October) of the Slovak National Canoe Slalom Federation team. The testing took place immediately after the end of the competition season and was a high priority for the athletes, as it, along with competition results, formed the basis for selection for a support project providing funding, coaching guidance, and optimal training conditions for the next season. All the data were collected from unique individuals over the course of three seasons. We included only one testing occasion/session per athlete in the dataset. Some athletes were tested over multiple years, but only the data from the year in which they achieved their best results at the World or European Championships were used in the analysis. The reason for collecting data over three seasons was to ensure a sufficient number of athletes in each category.

### Test protocols

#### On-water/flatwater testing

On-water testing was conducted at the Wild Water Complex (Čunovo, Slovakia), utilizing an artificial canal without water flow or underwater currents. Weather conditions were stable with wind up to 5 km/h and temperatures between 15°C and 23°C. The testing protocol was administered and carried out by a single experienced examiner, following a pre-established methodology.

Two specific tests, sprints with turns to both sides (SBS) and the 12 × 15 m an all-out shuttle test (12  ×  15 AOT), were performed on flatwater. These tests were designed and described by Vajda and Piatriková (5) and have been shown to exhibit a relationship with performance in canoe slalom on different water terrain grades (*r* = 0.638–0.909). In addition, both tests demonstrate high reliability, with an ICC of 0.98 for the SBS test and 0.96 for the 12 × 15 AOT test (12). A rest interval of 5 min was provided between tests, during which athletes engaged in light paddling.

All the tests started from a stationary position with the top of the boat in line between the poles. The subjects were instructed to build up to their maximal velocity and maintain their highest paddling velocity throughout the entire test. Each athlete performed their own warm-up routine and tested individually, so there was no racing/pacing with other individuals.

#### SBS test

The subjects paddled from the starting gate to the opposite gate, turned to the left side, paddled back to the starting gate, turned to the right side, and paddled to the opposite gate. The timing was started/stopped when the body of the subject crossed the yellow marker, which was placed in the middle of the course (12).

#### 12 × 15 AOT test

The athlete paddled from the starting gate to the opposite gate, turned to the left side, paddled back to the starting gate, turned to the right side, and paddled to the opposite gate. This was repeated six times. The timing started/stopped when the body of the athlete crossed the yellow marker, which was placed in the middle of the course (12).

Tests were recorded by a Panasonic HC-V800 video camera at 60 fps (Panasonic EP-K, Osaka, Japan) placed on a tripod, and the duration of each test was subsequently measured by Dartfish Pro video analysis software (Dartfish HQ, Fribourg, Switzerland).

#### Off-water testing

The off-water testing was conducted in cooperation with the National Sports Center at its facilities. The testing was carried out by an experienced diagnostic team, following the testing methodology protocol. The athletes were subject to a testing battery consisting of body composition analyses, power testing, and performance on a kayak/canoe ergometer.

### Anthropometric analyses

Body composition, including body height, body weight, and percentage of body fat, was measured. Body height was measured and determined to the nearest centimeter on an InBody BSM 170 digital free-standing stadiometer (InBody Co., Ltd., Cerritos, CA, USA). The body weight and percentage of body fat were measured with athletes wearing underwear using a Body 770 bioelectric impedance device (InBody Co., Ltd.). The athletes were asked not to eat for 2 h and drink for 1 h before the measurement. The devices were calibrated according to manufacturer guidelines and device specifications.

### Power output testing

Maximal power output (watts) and relative maximal power output (watts/kg) were measured in both prone bench press and bench pull exercises in a diagnostic series. Power output was measured by a Tendo Power Analyzer (Tendo Sports Machines, Trenčín, Slovakia) tethered to the barbell. At the beginning of the diagnostic series, the barbell was set at 40 kg for men and 20 kg for women, increasing by 2.5–5 kg in each subsequent set. Athletes were encouraged to push/pull the bar as fast as possible without releasing it or losing contact with it at the end of the concentric phase. Two minutes of passive recovery were allowed between each attempt with different loads. For each weight, the athletes performed two repetitions, and the attempt with the higher power output was recorded for further analysis. The diagnostic series was concluded when the athlete was no longer able to achieve a higher power output than in the previous set.

### Kayak/canoe ergometer testing

Testing on a kayak ergometer was performed on a Dansprint ergometer (Dansprint, Hvidovre, Denmark). The ergometer drag was adjusted for the body mass of each athlete according to the manufacturer's instructions to reproduce on-water conditions. The ergometer is normally set for the kayak category but can be extended for the canoe category. After 5 min of light paddling, the athletes performed an all-out 200 m run three times with a 4 min rest period between each run. During the rest period, the athletes engaged in light paddling. The time taken for the 200 m run and the mean mechanical power output for each 200 m attempt were measured and used for further analysis. In addition, the total time taken for all three attempts and the fatigue index during the tests were analyzed. The fatigue index was defined as the relative difference between the fastest and slowest 200 m performances, expressed as a percentage.

### Statistical analysis

All statistical procedures were performed using SPSS 23 (IBM, New York, USA), and data are presented as mean ± standard deviations (SD) and 95% confidence interval (95% CI). Normal distribution of the data was assessed both visually and using Shapiro–Wilk's test. Independent samples *t*-tests were used to analyze the difference between groups. Non-normally distributed data were analyzed with the Mann–Whitney *U*-test. Calculation of effect size was performed by using Hedge's g, where small (<0.3), medium (0.3–0.8), and large (>0.8) were used to describe the between-group differences. Statistical significance was accepted at *p* ≤ 0.05. Pearson correlation analysis was used to assess the linear correlation of the data. The relationship was assessed as small (*r* = 0.1–0.3), moderate (*r* = 0.3–0.5), large (*r* = 0.5–0.7), very large (*r* = 0.7–0.9), or nearly perfect (*r* > 0.9) (13).

## Results

All 34 athletes completed the testing over two consecutive days, and no data were excluded from the analyses. The general and anthropometric characteristics of the athletes are presented in Table 1.

Tables 2, 3 show the performance levels in the on- and off-water tests of each group. There were significant differences between the groups in the on-water tests in both the women's and men's categories (*p* < 0.05). In the women's categories, significant differences were found in the best time taken for 200 m, mean watts, and total 3 × 200 m time. In the men's categories, there were significant differences between all the parameters tested on the kayak ergometer, Pmax in bench press in watts, and relative Pmax in both bench press and pull. There were no significant differences in the other off-water parameters.

**Table 2:** Performance level in on-water testing.

Parameters		K1 women (*n* = 8)	C1 women (*n* = 8)	ES (g)	K1 men (*n* = 9)	C1 men (*n* = 9)	ES (g)
SBS (s)	X ± SD	15.69 ± 0.58	16.96 ± 0.47^a^	2.40	13.89 ± 0.48	15.86 ± 0.59^b^	3.66
	CI95%	15.19–16.18	16.55–17.36		13.52–14.27	15.40–16.31	
12 × 15 AOT (s)	X ± SD	104.00 ± 1.48	112.69 ± 2.42^a^	4.33	95.44 ± 1.91	105.64 ± 2.75^b^	4.30
	CI95%	102.75–105.24	110.66–114.72		93.96–96.91	103.52–107.76	

ES, effect size.

^a^ Significant difference between women’s categories (*p* < 0.05).

^b^ Significant difference between men’s categories (*p* < 0.05).

**Table 3:** Performance level in off-water testing.

Parameters		K1 women (*n* = 8)	C1 women (*n* = 8)	ES (g)	K1 men (*n* = 9)	C1 men (*n* = 9)	ES (g)
Best 200 m (s)	X ± SD	53.92 ± 3.02	68.97 ± 4.21^a^	4.10	45.82 ± 1.76	56.83 ± 2.50^b^	5.09
	95% CI	51.39–56.45	65.45–72.49		44.46–47.17	54.90–58.75	
Best 200 m (W)	X ± SD	168.63 ± 21.42	77.75 ± 19.21^a^	4.46	281.11 ± 29.36	156.33 ± 22.66^b^	4.75
	95% CI	150.71–186.54	61.68–93.82		258.54–303.68	138.91–173.76	
3 × 200 m total time (s)	X ± SD	167.63 ± 9.75	213.51 ± 14.08^a^	3.78	144.76 ± 5.20	176.13 ± 8.47^b^	4.46
	95% CI	159.48–175.79	201.73–225.29		140.76–148.76	169.61–182.65	
Fatigue index (%)	X ± SD	7.03 ± 4.60	6.08 ± 5.95	0.17	10.56 ± 5.71	6.27 ± 1.94	1.00
	95% CI	3.45–11.14	1.10–11.06		6.17–14.96	4.77–7.77	
Pmax bench press (W)	X ± SD	296.00 ± 42.07	288.88 ± 50.47	0.15	523.89 ± 59.30	436.00 ± 78.50^b^	1.26
	95% CI	260.83–331.17	246.68–331.07		478.30–569.48	375.66–496.34	
Pmax bench pull (W)	X ± SD	363.88 ± 59.59	329.75 ± 40.92	0.66	602.67 ± 47.21	551.67 ± 59.23	0.95
	95% CI	314.05–413.70	295.54–363.96		566.38–638.96	506.14–597.20	
Relative Pmax bench press (W/kg)	X ± SD	5.24 ± 0.95	5.09 ± 1.04	0.15	7.01 ± 0.68	5.82 ± 0.65^b^	1.78
	95% CI	4.44–6.03	4.22–5.96		6.48–7.54	5.32–6.32	
Relative Pmax bench pull (W/kg)	X ± SD	6.39 ± 0.93	5.80 ± 0.90	0.64	8.07 ± 0.59	7.40 ± 0.32^b^	1.41
	95% CI	5.61–7.17	5.05–6.56		7.61–8.53	7.15–7.66	

ES, effect size.

^a^ Significant differences between women’s categories (*p* < 0.05).

^b^ Significant differences between men’s categories (*p* < 0.05).

The relationship between the on- and off-water tests is presented in Tables 4, 5. Among the performance parameters, except for the fatigue index from the 3 × 200 m test, we found a significant relationship between SBS/12 × 15 AOT and all measured off-water test parameters, with correlation coefficients ranging from large to very large (*r* = 0.663–0.922).

**Table 4:** Relationship between anthropometric and on/off-water parameters.

Parameters	Best 200 m (s)	Best 200 m (W)	3 × 200 m total time (s)	Fatigue index (%)	Pmax bench press (W)	Pmax bench pull (W)	Relative Pmax bench press (W/kg)	Relative Pmax bench pull (W/kg)	12 × 15 AOT (s)	SBS (s)
Body height (m)	−0.520^b^	0.553^b^	−0.515^b^	0.169	0.660^b^	0.759^b^	0.254	0.427^a^	−0.441^b^	−0.429^a^
Body weight (kg)	−0.544^b^	0.600^b^	−0.556^b^	0.053	0.808^b^	0.862^b^	0.379^a^	0.485^b^	−0.478^b^	−0.472^b^
Body fat (%)	0.429^a^	−0.466^b^	0.408^a^	−0.273	−0.601^b^	−0.659^b^	−0.563^b^	−0.687^b^	0.445^b^	0.451^b^

^a^ Significant difference (*p* < 0.05).

^b^ Significant difference (*p* < 0.01).

**Table 5:** Relationship between on- and off-water performance parameters.

Parameter	Best 200 m (s)	Best 200 m (w)	3 × 200 m total time (s)	Fatigue index (%)	Pmax bench press (W)	Pmax bench pull (W)	Relative Pmax bench press (W/kg)	Relative Pmax bench pull (W/kg)
SBS (s)	0.851^a^	−0.902^a^	0.842^a^	−0.287	−0.748^a^	−0.704^a^	−0.735^a^	−0.727^a^
12 × 15 AOT (s)	0.884^a^	−0.922^a^	0.884^a^	−0.242	−0.705^a^	−0.693^a^	−0.663^a^	−0.700^a^

^a^ Significant difference (*p* < 0.01).

## Discussion

The purpose of this study was to investigate the level of measured parameters, calculate the differences between boat categories, and examine the relationship between on-water and off-water testing in canoe slalom. To the best of our knowledge, this is the first study to establish a relationship between on- and off-water performance in elite canoe slalom athletes. The main finding of the study shows that our selected off-water tests, focused on physical fitness, had a large to nearly perfect relationship with the flatwater tests. Our findings confirm existing practices of the coaches and athletes who regularly use the presented tests in testing and as training means in daily training practice.

### Anthropometric characteristics

In canoe slalom, common anthropometric characteristics have been observed according to the level of performance (7, 14). In the present study, no differences between boat categories in anthropometric parameters were found for the male and female athletes. Our findings are in line with Coufalová et al. (15), who did not observe differences between boat categories in anthropometric parameters in elite male and female canoe slalom athletes. They also provided average values for male canoe slalom competitors for body height (∼1.80 m), body weight (∼75 kg), and body fat (8% ± 3.2%). In our study, we found similar characteristics of the male athletes in the K1 category, namely body height = 1.80 ± 0.07 m, body weight = 74.81 ± 6.49 kg, and body fat = 9.13 ± 3.14%, and in the C1category: body height = 1.82 ± 0.03 m, body weight = 74.37 ± 6.50 kg, and body fat = 9.17 ± 1.37%. In the female categories, Coufalová et al. (15) provided average values for body height (∼1.64 m), body weight (∼59 kg), and body fat (17% ± 4.1%). Compared to these, our participants had similar characteristics in the K1 category, namely body height = 1.66 ± 0.07 m, body weight = 57.05 ± 5.60 kg, and body fat = 16.00% ± 5.22%, and in the C1 category: body = height 1.65 ± 0.07 m, body weight = 57.38 ± 7.76 kg, and body fat = 16.23 ± 2.95%. Our results should be interpreted with caution when applied to this age group. The findings are most likely applicable to athletes in a similar stage of development.

There is lack of evidence of the relationship between anthropometric parameters and on-water and/or off-water canoe slalom performance. Only one study, Messias et al. (16), conducted relationship analyses between on-water performance in simulated competition and anthropometric characteristics. The authors found a significant relationship between body fat (%) and on-water performance but not for body weight or body height. In the present study, we found the relationships between the anthropometric parameters and performance in on-water and off-water tests to vary from small to moderate. The important role of anthropometric parameters, especially body weight, is mostly related to the boats used in canoe slalom. The boats are designed and built to have optimal properties and functionality for athletes weighing between 50 and 80 kg. In the case of athletes with a weight of over 80 kg or under 50 kg, the desired buoyancy and maneuverability of the boat can be affected, which impacts proper technique, the speed of the boat, and other parameters.

### Power output testing

Power output was measured in two exercises that are regularly used in training programs of elite canoe slalom athletes (9). There is only one study, that of Bielik et al. (10), that has tested power output in bench press and bench pull in elite canoe slalom athletes. The authors used the same age groups and the same methodology to investigate power output in the bench press and bench pull as our study. In elite male athletes racing in both the K1 and C1 categories, Pmax was 530 ± 111 W in the bench press and 584 ± 83 W in the bench pull in the medalist group and 480 ± 66 W and 552 ± 56 W in the bench press and pull in the non-medalist group. In the present study, Pmax in the K1 men was 523 ± 59 W in the bench press and 602 ± 47 W in the bench pull, while the C1 men had 436 ± 78 W in the bench press and 551 ± 59 W in the bench pull. Bielik et al. (10) also calculated that the relative Pmax in the medalist group was 7.1 ± 1.4 W/kg in the bench press and 7.9 ± 0.8 W/kg in the bench pull, while in the non-medalist group, the values were 6.3 ± 0.7  and 7.3 ± 0.8 W/kg in the bench press and pull, respectively. In the present study, we found that the relative Pmax in the K1 men was 7.01 ± 0.69 W/kg in the bench press and 8.07 ± 0.59 W/kg in the bench pull, while in C1 men, the values were 5.82 ± 0.65 W/kg in bench press and 7.40 ± 0.32 W/kg in bench pull. In the female categories, there is no other study to compare our findings to. The differences between our findings and those of Bielik et al. (10) could be due to changes in the course setup in canoe slalom competition in the last 4 years. Since 2021, the courses have been shorter and demand more acceleration and deceleration during the run. These changes affected paddling techniques, requiring high-power strokes and a high number of powerful strokes during competition runs (17). Thus, these changes in technique are also mirrored in the physical fitness requirements of the elite athletes.

In addition, we found a significant relationship that ranged from large to very large (*r* = 0.663–0.735) between the flatwater tests and power output parameters. It should be noted that the flatwater tests are designed to reflect the performance-related physical fitness and physiological demands of a canoe slalom competition (5). Specifically, 12 × 15 AOT reflects the duration of the run and the number of turns performed at the competition. These turns require the acceleration and deceleration of the boat and require a high level of power output. Our findings reveal that these tests have a role in the ability of athletes to generate high-level power output. These findings should have deep implications for strength and conditioning training prescription.

### Kayak/canoe ergometer testing

In the literature, kayak ergometer testing in canoe slalom was utilized only to establish the VO2_max_ of the athletes (9). There are no published analyses of short-distance kayak ergometer testing in canoe slalom*.* In the present study, we analyzed all-out tests on kayak ergometers in three runs of 200 m distance with a 4-min rest interval. The differences between the groups reflect natural differences caused by the different categories, i.e., kayak and canoe. These differences were also found in the on-water tests. This information indicates practitioners should avoid comparing athletes from different categories. Furthermore, we found a significant relationship between SBS/12 × 15 AOT and time/mean watts production during the fastest 200 m run and total time for 3 × 200 m. These findings verify the good practice of coaches and athletes who use repeated 200 m runs as diagnostic tools and also as training means. The short 4-min rest interval was set so that the athlete could not fully recover between runs to be able to calculate the fatigue index between runs. However, there was no significant relationship between on-water performance and fatigue index.

The main limitation of our study is that we were not able to directly compare canoe slalom performance on whitewater to the off-water test performance, even though our designed and reliable tests demonstrate a strong relationship with canoe slalom performance on courses with various difficulty grades. Moreover, due to the limited number of participants in each category, the relationship between the on-water and off-water parameters was analyzed using pooled data instead of category-specific analyses. Future research should investigate the relationship between canoe slalom competition performance and off-water tests in more detail. Our study focused primarily on simple tests that can be easily implemented by coaches on a regular basis. In terms of strength and power production, future research could benefit from analyzing force and power output during flatwater and whitewater paddling using specialized paddle equipment. In addition, more detailed data on oxygen consumption and energy expenditure during both flatwater and whitewater runs would help to better understand the physiological mechanisms involved in performance.

## Conclusion

In previous studies, the relationship between on-water/flatwater tests and canoe slalom performance was verified. In this study, we demonstrated the relationship between selected on-water tests and off-water tests. The testing battery of off-water tests was assembled based on years of practical experience, with the selected tests serving not only as diagnostic tools but also as training instruments that are an integral part of the preparation of canoe slalom athletes. Our findings confirm existing practices. Based on the identified relationships, we recommend regular monitoring of the physical fitness levels of canoe slalom athletes using the described off-water tests. These tests can help identify the strengths and weaknesses of athletes, enabling coaches to optimize the training process.

## Data Availability

The raw data supporting the conclusions of this article will be made available by the authors, without undue reservation.
